# Junk Food Exposure Disrupts Selection of Food-Seeking Actions in Rats

**DOI:** 10.3389/fpsyt.2018.00350

**Published:** 2018-08-16

**Authors:** Alisa R. Kosheleff, Jingwen Araki, Linda Tsan, Grace Chen, Niall P. Murphy, Nigel T. Maidment, Sean B. Ostlund

**Affiliations:** ^1^Department of Psychiatry and Biobehavioral Sciences, Hatos Center for Neuropharmacology, Semel Institute for Neuroscience and Human Behavior, University of California, Los Angeles, Los Angeles, CA, United States; ^2^Department of Anesthesiology and Perioperative Care, University of California, Irvine, Irvine, CA, United States

**Keywords:** junk food, decision making, action selection, devaluation, outcome-specific PIT

## Abstract

There is growing evidence that repeated consumption of highly palatable, nutritionally poor “junk food” diets can produce deficits in cognition and behavioral control. We explored whether long-term junk-food diet exposure disrupts rats' ability to make adaptive choices about which foods to pursue based on (1) expected reward value (outcome devaluation test) and (2) cue-evoked reward expectations (Pavlovian-to-instrumental test). Rats were initially food restricted and trained on two distinct response-outcome contingencies (e.g., left press 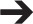 chocolate pellets, and right press 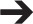 sweetened condensed milk) and stimulus-outcome contingencies (e.g., white noise 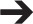 chocolate pellets, and clicker 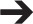 sweetened condensed milk). They were then given 6 weeks of unrestricted access to regular chow alone (controls) or chow and either 1 or 24 h access to junk food per day. Subsequent tests of decision making revealed that rats in both junk-food diet groups were impaired in selecting actions based on either expected food value or the presence of food-paired cues. These data demonstrate that chronic junk food consumption can disrupt the processes underlying adaptive control over food-seeking behavior. We suggest that the resulting dysregulation of food seeking may contribute to overeating and obesity.

## Introduction

The global obesity epidemic remains a serious health concern driven in part by changes in the global food supply—more cheap and processed foods are readily available in developed and developing countries than ever before [reviewed in Swinburn et al. (1)]. Many of these modern “convenience” and pre-packaged foods are not only cheap to procure, they also exploit our innate preferences for sugars, salts, and fats (2), causing cravings and excessive food intake in vulnerable individuals (3). Overeating and obesity seem to represent a failure of the multifaceted homeostatic processes that regulate energy balance and nutritional diversity (4), and there is growing evidence that repeated exposure to a junk-food diet can exacerbate the situation by further dysregulating feeding and food seeking. Specifically, animal studies have shown that poor diets (e.g., refined, high-fat, or high-sugar diets) can cause persistent aberrations in behavioral control and cognition, resulting in impulsive decision making (5, 6), motivational impairments (7), and altered food reward liking and craving (8–13).

Experience with such diets also appears to disrupt the way in which rats select food-seeking actions based on the expected value of food rewards (14–16). For instance, normal chow-fed rats will inhibit their pursuit of a palatable food reward (e.g., lever pressing for sugar solution) if they have been given the opportunity to feed to satiety on that particular food prior to testing (a method of reward *devaluation*) (17, 18). In contrast, rats that have been maintained on a diet that includes sugary or fatty foods seem to lose this ability to control their food seeking, in that they are more likely to seek out a food upon which they have been sated, relative to controls (14–16, 19). Interestingly, such diets appear to cause behavioral insensitivity to food devaluation regardless of whether that behavior is the product of instrumental (14–16) or Pavlovian conditioning (19). While this suggests a general impairment in behavioral control, the specific causes of this inflexibility are not well understood. One possibility is that rats with repeated experience consuming palatable junk foods come to evaluate foods differently, and may be less sensitive to specific satiety or similar methods of devaluation. Given evidence for junk food-induced impairments in learning and memory (20–22), junk-food diets may also interfere with more cognitive aspects of behavioral control, such as the ability to retrieve and make use of specific action-outcome or stimulus-outcome mappings. This latter hypothesis predicts that junk-food diets should impair control over specific food-seeking actions even when food values are not the primary basis for decision-making.

We tested this hypothesis by comparing the effect of junk-food diet exposure on rats' ability to select actions based on outcome value and cue-evoked outcome expectations. Environmental cues that signal foods (e.g., packaging and jingles) can elicit strong food cravings (23, 24), which cause some individuals to overeat (25–27). This influence can be modeled in rats using the outcome-specific Pavlovian-to-instrumental transfer (PIT) paradigm, which assays the tendency for a cue that has become associated with a specific food to selectively trigger the performance of an action that produces that food, relative to another action that produces a different food outcome (28, 29). The current study investigated the effects of extended junk-food exposure on rats' ability to choose between instrumental food-seeking actions using the satiety-based food devaluation and outcome-specific PIT tests. Importantly, recent studies have shown that the pattern in which palatable food diets are consumed influences their ability to dysregulate behavior. For instance, rats given intermittent access (e.g., 1–2 h per day) to sugar or other junk foods develop a binging pattern of consumption (30, 31) and display addiction-like behaviors not seen in rats given *ad libitum* access (24 h per day) to such diets or in control rats (32, 33). We therefore included an assessment of the effects of both intermittent and *ad libitum* access to junk food on decision making in the current study.

## Methods

### Subjects and apparatus

Adult male Sprague-Dawley rats (*n* = 24; 8 weeks old at the beginning of food restriction) were pair-housed for the duration of the experiment. Rats were food restricted to ~ 85% of their projected free-feeding body weight during initial behavioral training. All behavioral training took place in sound- and light-attenuating operant chambers (Med Associates, East Fairfield, VT). Each chamber was equipped with two retractable levers, a white noise generator, a clicker audio generator, a pellet dispenser that delivered chocolate flavored pellets (45-mg, Bio-Serv, Frenchtown, NJ) and an infusion pump that delivered a 0.1 ml infusion of 50% sweetened condensed milk solution/H_2_O (SCM) into a food cup. All experimental procedures were approved by the UCLA Institutional Animal Care and Use Committee and were in accord with the National Research Council Guide for the Care and Use of Laboratory Animals. The experimental timeline is summarized in Table 1, and described in detail below.

**Table 1:** Experimental timeline.

**Phase**	**Duration**	**Procedure**
Pavlovian conditioning	8 days	Cue_1_ → Outcome_1_ Cue_2_ → Outcome_2_
Instrumental training	11 days	Response_1_ → Outcome_1_ Response_2_ → Outcome_2_
Diet exposure	6 weeks	Control, intermittent or *ad libitum* exposure
Mild food restriction	3 days	14 h chow per day
Outcome devaluation test 1	1 day	Sated on Outcome_1_, both levers extended, no outcomes
Outcome devaluation test 2	1 day	Sated on Outcome_2_, both levers extended, no outcomes
PIT test	1 day	Cue_1_ and Cue_2_ present, both levers extended, no outcomes

### Pavlovian conditioning

Behavioral training was modified from protocols previously used in our laboratory (34, 35). Rats received 8 daily sessions of Pavlovian conditioning, where each of two auditory cues (white noise or clicker) was consistently paired with one of the outcomes. For half of the subjects, the clicker was paired with chocolate pellets, and the white noise was paired with SCM, whereas the other half of the subjects received the opposite stimulus-outcome pairings. Each training session consisted of 8 total trials, during which each stimulus was presented 4 times. Each trial (i.e., cue) lasted 2 min, during which the corresponding outcome was delivered on a random time (RT) 30 s schedule. Each trial was separated by a variable 5-min inter-trial interval (range = 4–6 min). To measure conditioned responding, we calculated the rate of food cup entries during pre-cue periods and during the initial period of the cues prior to the first reward delivery on that trial.

### Instrumental training

Rats received 11 days of instrumental training, with two sessions conducted each day, separated by at least 20 min. Access to the left and right lever was alternated between the 2 sessions. In the first session, rats were trained that pressing the left (or right) lever would result in delivery of the pellet (or SCM) reward (counterbalanced), with the second session providing training with the other response-outcome contingency. Each day, the session order was reversed from the previous day. During the first 2 days of training, each lever-press response was continuously reinforced with the appropriate food outcome. The reinforcement schedule was then changed to random ratio 5 (RR-5) for days 3–4, RR-10 for days 5–6, RR-15 for days 7–8, and RR-20 for days 9–11.

### Junk-food diet

Following training, rats were assigned to one of three diet groups: Controls, Intermittent, or *ad libitum*. During this time, all rats had continuous access to chow and water in their home cages, while the two treatment groups (Intermittent and *ad libitum*) also received access to two junk foods (one sweet, one savory) each day for either 1 h only (Intermittent group) or for 24 h (*ad libitum* group), using protocols commonly used to model binge and chronic overeating, respectively (10, 11, 36). Different junk foods were made available each day. Junk foods consisted of Hershey's chocolates, Kit Kats, Chips Ahoy cookies, Oreo cookies, shelf-stable sugar-coated donuts, shelf-stable brownies, Ritz crackers, Cheetos, Doritos, bagels, hot dogs, and cheddar cheese. Diet exposure was continued for 6 weeks, after which point rats were maintained on 14 h access to standard laboratory chow only (i.e., 10 h food deprivation) to maintain mild food deprivation for behavioral testing. Importantly, because our intent was to assess the impact of junk-food exposure on the expression of food-seeking behavior, and not on learning about such actions, we conducted these tests in extinction (no reinforcement), without any further post-diet exposure retraining.

### Outcome devaluation testing

After 3 days of mild food restriction, rats were given two sessions of outcome devaluation testing (devaluing one outcome for test 1, and the other for test 2, 48 h apart). Here, we evaluated how diet exposure influenced rats' ability to adapt their choice between food-seeking actions after being selectively sated on one of the two food rewards. Immediately preceding each test, rats were singly housed with water *ad libitum*, and given unrestricted access to either chocolate pellets or SCM for 1 h (counterbalanced). This sensory-specific satiety procedure is used to temporarily reduce the incentive value of the food (i.e., devaluing), while leaving the incentive value of the alternate food unaffected. Immediately after this, rats were placed in the operant chambers for a 5-min choice extinction test during which both levers were extended, but no auditory cues were presented nor were any outcomes delivered. Lever presses and food cup entries were continuously recorded. Forty-eight hours later, rats were given a second outcome devaluation test using the opposite outcome (i.e., if test 1 used pellets, test 2 used SCM).

### Pavlovian-to-instrumental transfer testing

Forty-eight hours after the second devaluation test, rats underwent a PIT test in order to assess the effects of junk-food exposure on the outcome-specific influence of food-paired cues on food-seeking behavior. Both levers were inserted into the chamber for the duration of the session. Lever presses and food cup entries were continuously recorded, but no outcomes were delivered. During the PIT test, after an initial 5 min, each cue was presented for 2 min non-contingently (i.e., cue onset and offset occur regardless of lever pressing) on 4 separate trials (8 trials total). A pseudorandom (ABBABAAB) trial order was used, with trials separated by a fixed 5-min interval.

### Data analysis and statistics

Because rats were pair-housed during the junk-food exposure phase, junk food consumption was recorded on a per-cage basis. Per day consumption (in kCal) was normalized to (divided by) the total body weight (kg) of each cage's rats. Consumption is reported as the total calories consumed in each 24 h cycle, as well as each 1 h binge session (when each day's new foods were introduced). Calories obtained from food (chocolate pellets or SCM) consumed prior to the outcome devaluation test are analyzed and presented as kCal normalized to (divided by) rat body weights (kg).

Devaluation test data and PIT lever presses at baseline and food cup entries are reported as total counts, but statistical analyses were conducted on the square root transformations. For the PIT test, we averaged across individual trials. The influence of cue presentations on lever-press performance are reported using an elevation ratio that was computed separately for each action: i.e., [Same cue/(pre-cue + Same cue)] and [Different cue/(pre-cue + Different cue)]. Thus, a ratio score above 0.5 indicates that an action was performed at a higher rate during the cue than during the pre-cue (baseline) period. These data are reported as an elevation ratio, but statistical analyses were conducted on the logit transformation of these data. Main effects, interactions and *post-hoc* tests were defined as statistically significant when *p* < 0.05. An exception is in the case of multiple (greater than two) *post-hoc* comparisons, where criteria for significance was corrected using Holm's sequential Bonferroni correction (37, 38), e.g., in a family of 3 comparisons, at least one comparison must meet an alpha criterion of *p* ≤ 0.0167 (i.e., 0.05/3), while a second comparison must meet an alpha criterion *p* ≤ 0.025 (i.e., 0.05/2), while the last comparison must meet a criterion of *p* ≤ 0.05 (0.05/1), in order for each effect to be considered significant. Therefore, when applying Holm-corrected significance criteria, *p-*values are expressed to 3 decimal places where necessary. All data were analyzed using SPSS (IBM, Armonk, NY), and are expressed as means ± standard error of the mean (SEM).

## Results

### Behavioral training

Behavioral training occurred in two phases: Pavlovian conditioning and instrumental lever press training. Each phase was analyzed for potential pre-existing differences between future diet group assignments.

### Pavlovian conditioning

To assess conditioned responding going into the diet-exposure phase, we analyzed the difference in rate of food cup entries during cue presentations (between cue onset and first reward delivery) relative to the pre-cue period for the last 3 days of Pavlovian conditioning (averaged). A univariate analysis of diet failed to reveal any effect of future diet assignment on conditioned responding [*F*_(2, 21)_ = 0.21, *p* = 0.82] and a one-sample *t*-test (vs. 0) confirmed that conditioned food-cup entries were significantly elevated during cue presentations [*t*_(23)_ = 21.35, *p* < 0.001].

### Instrumental training

All groups readily learned to perform both lever-press actions. To assess lever-press responding going into the diet exposure phase, we analyzed the mean response rate during the last 3 days of instrumental training (averaged). An outcome (pellet vs. SCM) × diet rmANOVA revealed a significant main effect of food type [*F*_(1, 21)_ = 69.17, *p* < 0.001), with rats pressing at a higher rate for pellets (mean 42.89 ± 0.42 SEM) vs. SCM (mean 22.10 ± 0.39), but failed to show a main effect of future diet assignment on lever press rate [*F*_(2, 21)_ = 1.05, *p* = 0.37), or a diet × food type interaction [*F*_(2, 21)_ = 0.91, *p* = 0.42].

### Food consumption during the junk-food exposure phase

**Figure 1:**
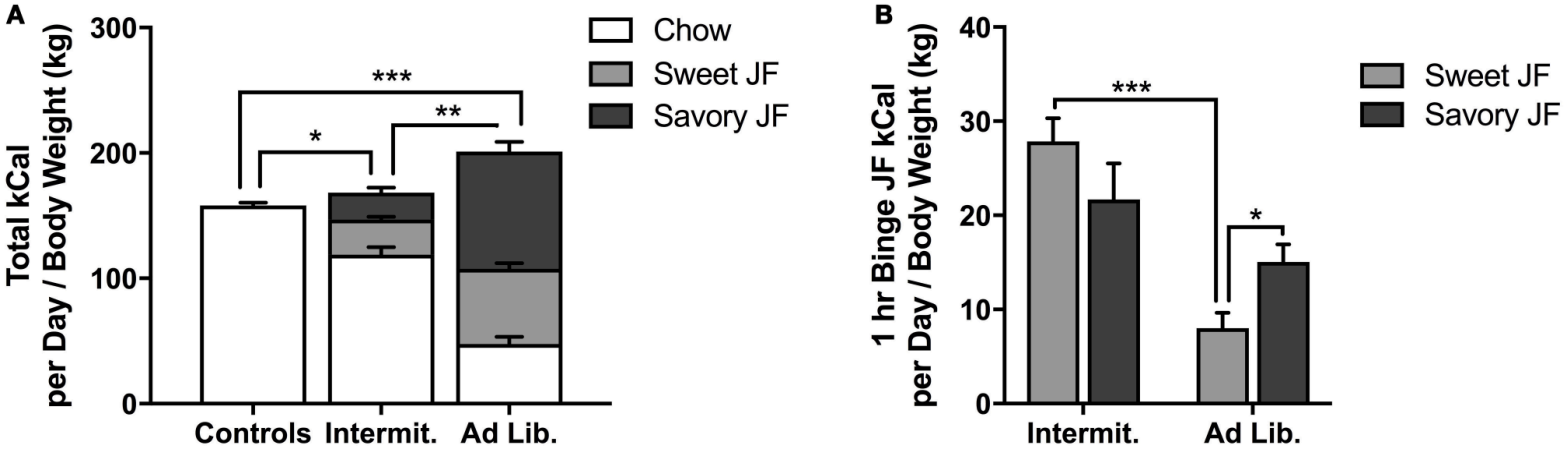
Junk Food (JF) Consumption. **(A)** Average total calories (kCal) consumed per day (adjusted to rat weight in kg), separated by chow, sweet junk foods, and savory junk foods. *Ad libitum* rats consumed significantly more calories per day than both Controls or Intermittent rats, while Controls consumed the fewest calories. **(B)** Average total junk food calories consumed during the 1 h binge feeding period, separated by sweet and savory junk foods. Intermittent rats consumed similar amounts of calories from sweet and savory junk foods, while *ad libitum* rats consumed significantly more calories from savory than sweet junk foods. Intermittent rats consumed significantly more calories from sweet junk foods than *ad libitum* rats during this period. Means ± SEM. ^*^p < 0.05, ^**^p < 0.01, ^***^p < 0.001.

Food consumption during the junk-food exposure phase is reported on a per-cage basis (in kCal), normalized to (divided by) the total body weight (kg) of each cage's rats. Data are shown for the full 6 week period, divided by 42 to get a per-day average. An ANOVA of daily consumption revealed a significant effect of diet [*F*_(2, 9)_ = 37.37, *p* < 0.001; Figure 1A), and Holm-corrected independent samples *t*-tests found that *ad libitum* rats consumed more calories than Intermittent rats [*t*_(6)_ = 5.47, *p* = 0.002] and Controls [*t*_(6)_ = 7.27, *p* < 0.001]. Intermittent rats also consumed more calories than Controls [*t*_(6)_ = 3.21, *p* = 0.018].

A rmANOVA of sweet vs. savory foods consumed during the 1 h binge period (Figure 1B) revealed a significant effect of diet [*F*_(1, 6)_ = 18.68, *p* = 0.005; where Intermittent rats consumed significantly more junk food than *ad libitum* rats), and a significant diet × food interaction [*F*_(1, 6)_ = 10.87, *p* = 0.016]. Independent samples-*post hoc* tests revealed that Intermittent rats consumed more sweet foods during this time than *ad libitum* rats [*t*_(6)_ = 6.71, *p* < 0.001], but not savory foods [*t*_(6)_ = 1.56, *p* = 0.17]. Paired *t*-tests (sweet vs. savory) failed to reveal a difference in sweet vs. savory consumption among Intermittent rats [*t*_(3)_ = 1.80, *p* = 0.17], but did reveal that *ad libitum* rats consumed more savory foods than sweet [*t*_(3)_ = 3.40, *p* = 0.04]. The main effect of diet suggests that Intermittent rats consumed junk foods in a binge-like pattern, distinct from the *ad libitum* rats (30, 31).

### Change in body weights

**Figure 2:**
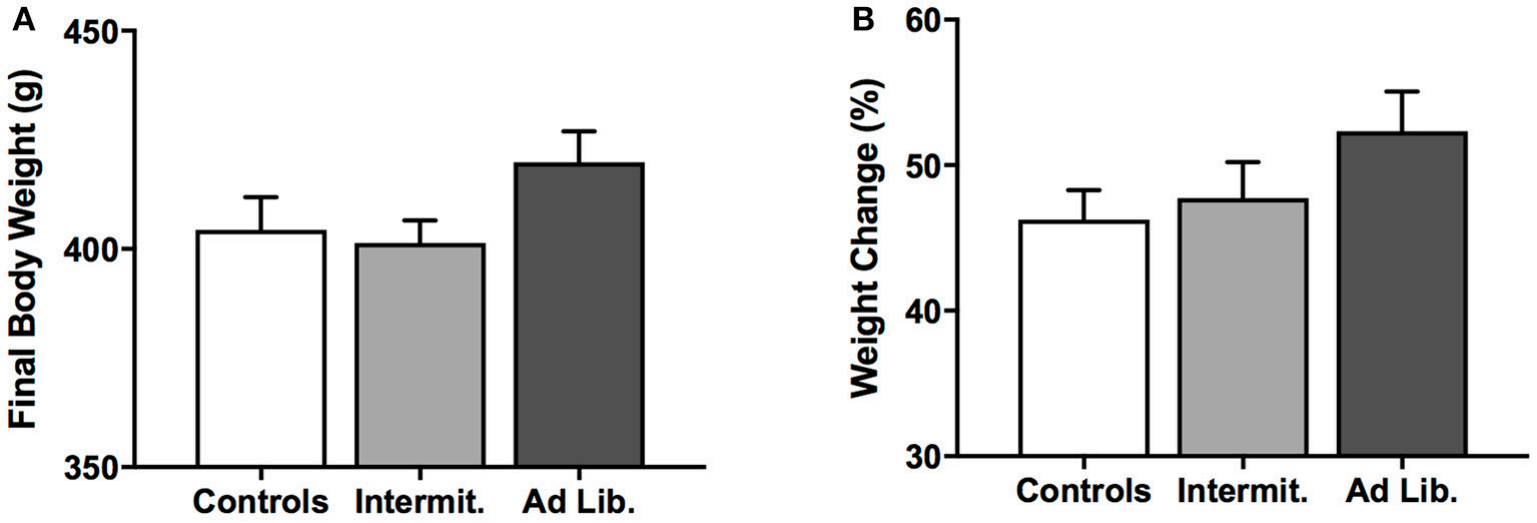
Changes in body weight. **(A)** Final body weights after the junk-food exposure phase. **(B)** Weight gained during the junk food exposure phase, expressed as a percentage of individual starting weights. There was no significant difference between diet groups in final body weight or weight gain as a percentage of initial body weight, although there was a trend for *ad libitum* rats to weigh more than Intermittent rats at the end of the junk-food exposure phase. Means ± SEM.

All rats gained weight during the 6-week period of junk-food exposure. Although *ad libitum* rats tended to weigh more than the other groups immediately after the junk-food exposure phase, ANOVAs failed to reveal any effect of diet on final body weight [*F*_(2, 21)_ = 2.22, *p* = 0.13; Figure 2A] or weight gained as a percentage of initial body weights on the first day of junk-food exposure [*F*_(2, 21)_ = 1.72, *p* = 0.20; Figure 2B].

### Food consumption during the consumption phase of the devaluation test

**Figure 3:**
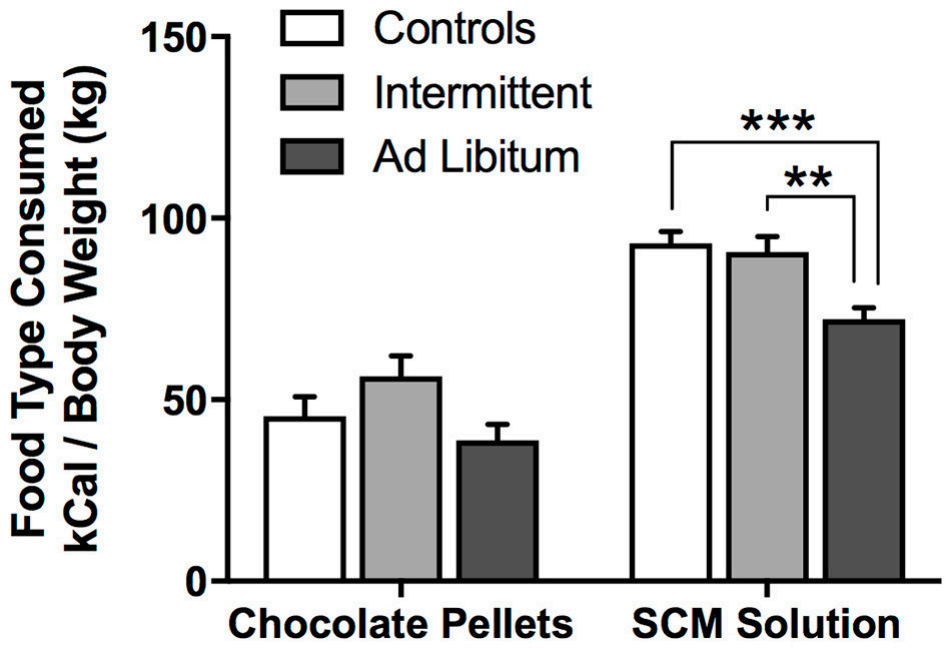
Outcome calories consumed during the pre-feeding phase of the devaluation test. Immediately prior to the devaluation test, rats were given 1 h access to either chocolate pellets or SCM solution. Their food consumption during this period is presented here as calories (kCal) adjusted to body weight (kg). Rats in the *ad libitum* group consumed the least amount of each outcome. Means ± SEM. ^**^p < 0.01, ^***^p < 0.001.

To assess the effect of junk-food diet exposure on food consumption (chocolate pellets or SCM) during the specific-satiety treatment, we conducted a diet × food type rmANOVA (Figure 3). This analysis revealed a significant effect of food type [*F*_(1, 21)_ = 203.32, *p* < 0.001], where all groups consumed more calories from SCM than from pellets. This analysis also revealed a significant main effect of diet [*F*_(2, 21)_ = 6.33, *p* = 0.007), and a trend for a significant diet × food type interaction [*F*_(2, 21)_ = 2.96, *p* = 0.07]. Between-groups *post-hoc* tests for each food type revealed that *ad libitum* rats consumed fewer SCM calories than Intermittent rats [*t*_(14)_ = 3.50, *p* = 0.004] and Controls [*t*_(14)_ = 4.61, *p* < 0.001]. *Ad libitum* rats also appeared to consume fewer calories from pellets [*t*_(14)_ = 2.51, *p* = 0.025], though this did not reach Holm-corrected criteria for significance. Importantly, experimenters visually confirmed that all rats spent a substantial amount of time actively consuming food during the early part of the feeding session, and showed relatively little interest in the food by the end of the session, suggesting that they were all sated on that food by the end of this treatment.

### Outcome devaluation testing

**Figure 4:**
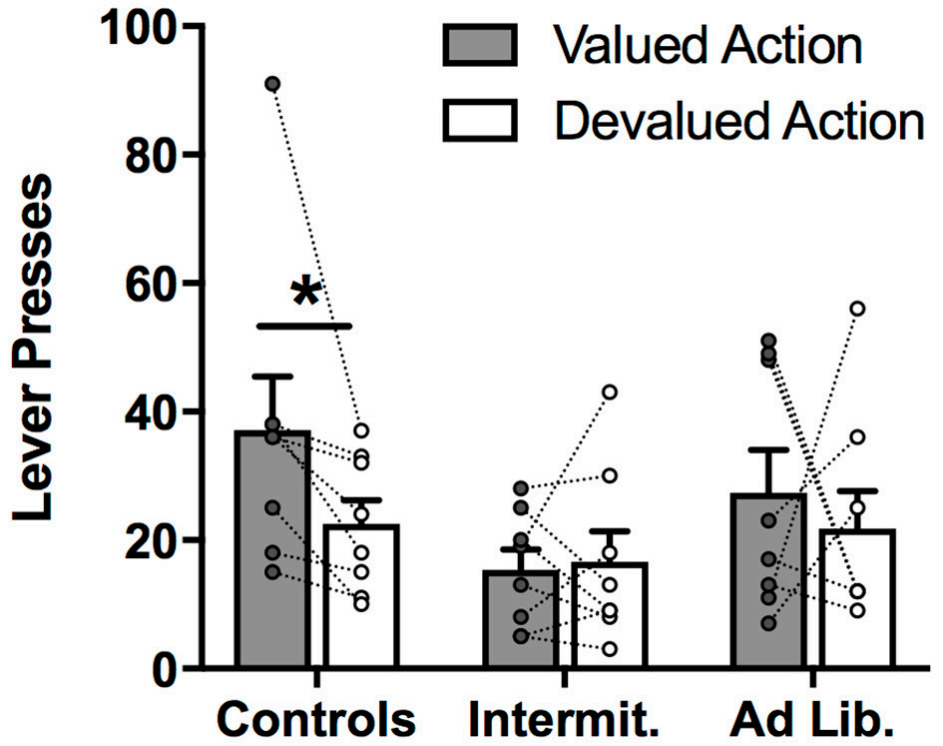
Outcome devaluation test. Lever presses for the lever associated with either a valued or devalued outcome. Control rats pressed more on the valued versus devalued lever, while both junk food groups failed to show a preference for one action over the other. Means ± SEM, with individual points and repeated-measures lines. ^*^p < 0.05.

A diet × action (valued vs. devalued) rmANOVA conducted on the number of presses performed (Figure 4) failed to detect a significant main effect of action (valued vs. devalued) [*F*_(1, 21)_ = 2.04, *p* = 0.17], or a diet × action interaction [*F*_(2, 21)_ = 0.88, *p* = 0.43], although there was a trend toward a significant main effect of diet [*F*_(1, 21)_ = 3.18, *p* = 0.06], with junk-food groups, and particularly the intermittent access group, showing generally less food seeking than the control group. Despite the near-significance of the diet effect, the overall amount of pressing did not significantly differ across diet groups after Holm-correcting for multiple comparisons (*p*'s > 0.05).

Based on previous studies showing diet-induced impairment in sensitivity to outcome devaluation (14, 16, 19), we conducted *a priori* paired *t*-tests (valued vs. devalued; Holm-corrected) to determine whether rats in the junk-food groups tested here also exhibited insensitivity to outcome devaluation. Wilcox [(39), p. 36] advises that *a priori*, planned comparisons appropriately corrected for Type I error as a result of multiple comparisons do not require a significant *F* test: “in terms of controlling the Type I error probability, most multiple comparison procedures should be used regardless of whether the *F-*test is significant,” and suggests the Bonferroni-adjusted procedure as appropriately conservative. As expected, the Control group showed normal sensitivity to devaluation, with rats significantly reducing performance of the action associated with the devalued outcome, relative to the other action [*t*_(7)_ = 3.22, *p* = 0.015; with the high responder on “valued action” removed: *t*_(6)_ = 3.56, *p* = 0.012; Holm-corrected). In contrast, neither of the junk-food diet groups showed a significant devaluation effect [Intermittent: *t*_(7)_ = 0.11, *p* = 0.92; *ad libitum*: *t*_(7)_ = 0.51, *p* = 0.63]. These results suggest that, while our Control rats were able to appropriately reduce responding for the devalued outcome, rats in the junk-food conditions were impaired in this aspect of action selection.

It is important to note that there was more variability in the degree of sensitivity to outcome devaluation in the junk-food groups than in Controls: while all Control rats showed at least some degree of devaluation, this was not the case with junk-food groups, as many of these rats failed to show an overall preference for the valued action, relative to the devalued action. Specifically, whereas all 8 rats in the Control group performed the devalued action at a lower rate than the valued action, only 4 out of 8 rats in the Intermittent group, and only 5 out of 8 rats in the *ad libitum* group showed this effect.

### Outcome-specific pavlovian-to-instrumental transfer testing

**Figure 5:**
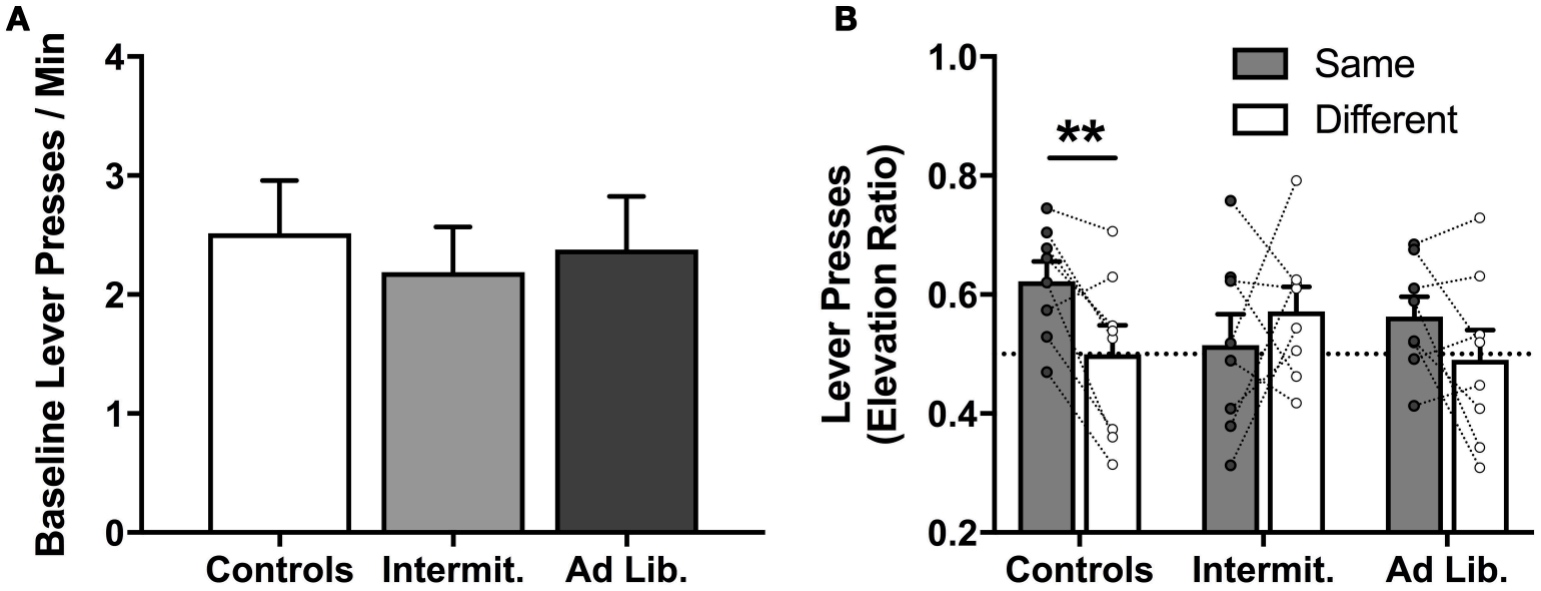
Lever presses during the PIT test. **(A)** Groups did not differ on pre-cue (baseline) instrumental response rates, suggesting junk-food exposure did not alter general instrumental responding. **(B)** Lever pressing in response to cues, presented as an elevation ratio from pre-cue responding [cue / (pre-cue + cue)]. Only rats from the Control group successfully used previously learned stimulus-outcome and action-outcome associations to guide action selection, increasing their responding on the “Same” lever significantly more than on the “Different” lever. Means ± SEM, **(B)** with individual points and repeated-measures lines. ^**^p < 0.01.

**Figure 6:**
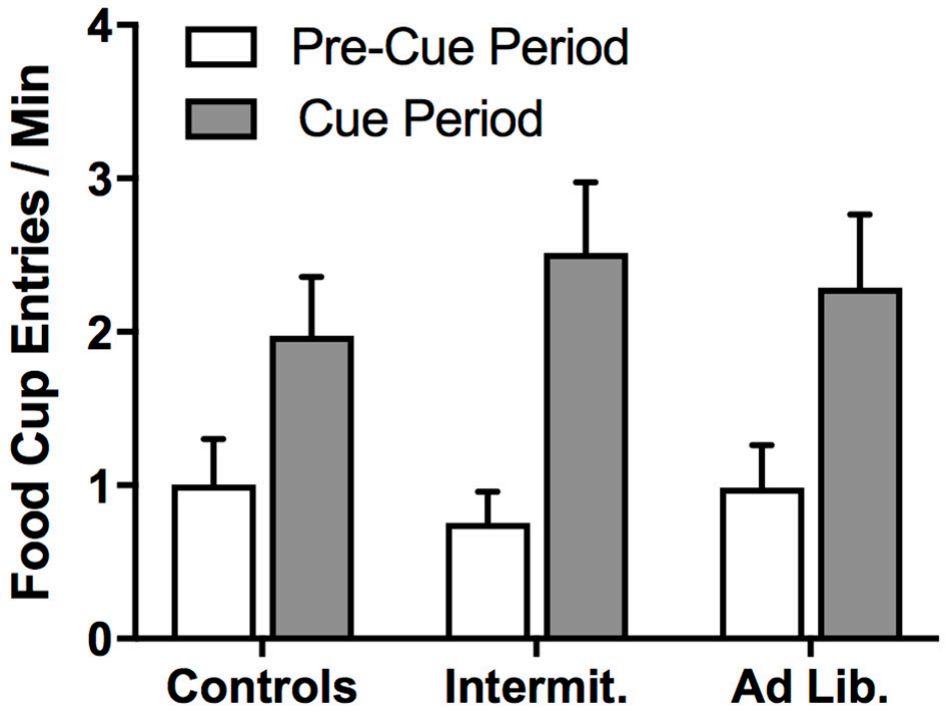
Food cup entries during the PIT test. All groups showed similar levels of conditioned approach food cup entries, suggesting that diet did not alter food cup approach in response to cues. Means ± SEM.

After 1 day off, we conducted a PIT test to assess whether junk-food exposure alters rats' ability to use food-paired cues to invigorate food-seeking actions based on a shared outcome representation; i.e., that a cue predictive of pellets motivates pressing for pellets (Same) and not SCM (Different), and vice versa. A univariate analysis of pre-cue, baseline lever pressing (both levers combined) found that diet had no effect on uncued food seeking [*F*_(2, 21)_ = 0.16, *p* = 0.85] (Figure 5A). The influence of the food-paired cues on lever-press rates (elevation ratio) was analyzed using a diet × action (Same vs. Different) rmANOVA, which did not detect a significant effect of action [*F*_(1, 21)_ = 2.62, *p* = 0.12] or diet [*F*_(2, 21)_ = 0.22, *p* = 0.80], but did detect a significant diet × action interaction [*F*_(2, 21)_ = 3.65, *p* = 0.04] (Figure 5B). Holm-corrected paired *t*-test comparisons revealed a significant difference in levels of responding on the Same vs. Different action for Control group [*t*_(7)_ = 3.75, *p* = 0.007], but no such effect was detected for the Intermittent [*t*_(7)_ = 0.89, *p* = 0.40] or *ad libitum* junk-food groups [*t*_(7)_ = 1.66, *p* = 0.14]. These results demonstrate that extended access to a junk-food diet disrupted rats' tendency to use cue-elicited food expectations to guide their food-seeking behavior. A diet × cue (pre-cue vs. cue) rmANOVA performed on the rate of food cup entries (Figure 6) during the PIT test revealed a significant effect of cue [*F*_(1, 21)_ = 83.75, *p* < 0.001], where all rats checked the food cup more during cue presentations, but found no effect of diet [*F*_(2, 21)_ = 0.02, *p* = 0.98] or diet × cue interaction [*F*_(2, 21)_ = 2.06, *p* = 0.15].

## Discussion

We report that junk-food exposure profoundly impacts rats' decisions about which foods to pursue based on both the perceived value of the food and predicted availability of the food. Rats given 6 weeks of junk-food exposure tended to disregard the current value of potential foods when engaged in food-seeking behavior (outcome devaluation test; Figure 4), and also failed to use cue-elicited food expectations to guide their selection of actions based on the specific foods they produce (outcome-specific PIT test; Figure 5B). Because animals were trained with these stimulus-outcome and action-outcome contingencies *prior* to junk-food exposure, these effects reflect a deficit in retrieval and/or decision-making processes, rather than an impairment in the initial acquisition of such relationships. It is also notable that these deficits in action selection occurred even though junk-food exposure had no effect on baseline levels of instrumental food seeking (lever pressing; Figure 5A) and anticipatory food-cup approach behavior during the PIT test (Figure 6), and only modest (and statistically insignificant) effects on overall levels of instrumental food seeking during the outcome devaluation test. This suggests that, while junk-food-fed rats may have been more sensitive to the nonspecific, response-suppressing effects of our satiety manipulation, the impact of diet on decision making was not secondary to more basic and wide-ranging effects on behavioral performance.

Rats with a history of junk-food exposure were impaired in using specific-satiety-induced changes in the value of food rewards to guide their selection of specific food-seeking actions. This result is in line with several previous reports (14–16, 19), but not others. In particular, Tantot et al. (15) recently reported that rats exposed to a high-fat diet exhibit insensitivity to devaluation only if they are trained on a random interval schedule. Such training tends to promote a transition to habitual reward-seeking behavior, which is, by definition, performed without consideration of its consequences. In contrast, such a diet was not found to impact behavior for rats that were trained on a random ratio schedule, which tends to encourage the use of a goal-directed behavioral strategy that is highly sensitive to outcome devaluation. This might suggest that energy-dense and/or nutritionally poor diets impact sensitivity to devaluation by facilitating transition to habitual responding, but only in situations that would normally encourage such a transition in behavioral control. In contrast to this view, however, we found that long-term exposure to a diverse, highly-palatable junk-food diet, either via intermittent or *ad libitum* access, interfered with rats' ability to choose between two distinct food-seeking actions based on a selective reduction in the value of one specific food outcome, a task which is known to strongly discourage habitual performance, even after over-training (40, 41). Thus, our finding is unlikely to be related to a facilitation of normal habit formation, particularly given that rats received no further lever-press training after the diet exposure phase. Instead, they may have been impaired in adaptively updating the value of potential food goals based on their specific-satiety experience, a process referred to as instrumental incentive learning (42). However, our finding that these rats were also impaired in using specific cue-elicited food expectations when deciding between food-seeking actions, even though this did not require them to reassess the value of potential food goals, suggests that they had difficulty retrieving and/or using the cue-outcome or action-outcome associations encoded more than 6 weeks prior. Indeed, the considerable amount of time between training and testing is an important feature of this study: we explicitly avoided retraining rats after diet exposure to prevent junk food-induced alterations in learning (20–22, 43–45) from contaminating our assessment of decision-making.

It is perhaps surprising that this aspect of decision-making is compromised following a simple change in diet given that rats exposed chronically to cocaine show relatively normal sensitivity to outcome devaluation on this same goal-directed action selection task (46). It may be that experiences consuming junk foods are particularly relevant and, therefore, have a lasting impact on how we decide which foods are worth pursuing. We suggest that the tendency for junk-food diets to disrupt action selection for food reward may result from diet-related learning rather than a permanent and unconditional cognitive impairment. For instance, there is growing evidence that rats' capacity for goal-directed action selection for food rewards is acutely disrupted in the presence of contextual cues that signal access to junk food (46), an effect that has also been shown with drug-paired cues [either methamphetamine (47) or alcohol (48)]. Thus, it is possible that rats given extensive junk-food exposure acquired a persistent—and perhaps less cue-dependent—expectation of having access to junk food, and that this influenced the way they made decisions about other food-seeking actions. This might also explain why we observed considerable variability across rats in the effect of junk-food exposure on devaluation performance, given that expectations about junk food likely change over time in a manner that varies with individual experiences.

In a related study, we found that rats given intermittent junk-food access were also impaired in a distinct, nonspecific version of the Pavlovian-to-instrumental transfer task (8). In that study, rats that had been given intermittent access to a junk-food diet showed indiscriminant instrumental food-seeking behavior in response to both food-paired and unpaired cues, unlike chow-fed rats, which showed a selective increase in responding during trials with the food-paired cue. We hypothesized that this indiscriminate, over-generalization in responding to environmental cues may be due to sensitization of mesolimbic dopamine transmission, which can occur after intermittent (32, 49–51) and, to a lesser extent, chronic access (52) to junk-food diets. In support of this idea, dopamine signaling is strongly implicated in cue-motivated food seeking (53–55) and increases in cue-elicited mesolimbic dopamine release have been implicated in the facilitation of PIT performance in cocaine-experienced rats (54).

However, it is not clear how the effects of junk-food exposure on non-specific and outcome-specific forms of PIT compare. Although junk-food exposure disrupted the influence of food-paired cues on food-seeking behavior in the current study, it did not do so by increasing rats' willingness to respond to those cues, but instead tended to disrupt the behavioral influence of such cues, particularly their ability to guide action selection to facilitate pursuit of the expected food outcome. Because the non-specific and outcome-specific forms of PIT are mediated by dissociable neural systems (56, 57), it is possible that junk-food exposure impacts these behavioral phenomena in fundamentally different ways. For instance, it is unlikely that the effects of junk food on outcome-specific PIT are related to a change in dopamine signaling given evidence that the expression of this behavioral effect is relatively insensitive to dopamine receptor blockade (58), unlike the general or non-specific form of PIT (59–61). That being said, the effects of junk food on specific and non-specific forms of PIT may be related to a broader disruption in the use of stimulus-food associations to guide behavior (14–16, 19). Indeed, high-fat diets have also been shown to impair conditional discrimination learning, leading to excessive cue-triggered lever-press performance when this behavior is not reinforced (62). Further, in a hippocampal-dependent negative feature discrimination problem, junk-food exposed rats were unable to learn that the presence of a discrete cue signaled the omission of a reward, increasing appetitive responding even on unreinforced trials (63). Such data offer further support that junk-food consumption may impair the accurate and adaptive use of environmental cues to guide reward-seeking behavior.

It is important to note that although junk-food exposure disrupted rats' ability to adjust their selection of food outcomes based on changes in food value or cue-elicited food expectations, they did not exhibit excessive levels of food-seeking behavior. If anything, rats with a history of junk-food exposure showed at least a trend toward suppression of food seeking during the outcome devaluation test, in line with previous studies reporting deficits in reward processing in rodents exposed to poor quality and junk-food diets, as indicated by increased brain self-stimulation thresholds (10), decreased conditioned place preference for amphetamine (64), decreased ethanol consumption (65), and decreased motivation for food reward on a progressive ratio task (7). What is interesting is that these rats seem to pursue food in a less discerning manner, engaging in food seeking without considering the consequences of their actions. Such findings may be related to a growing body of evidence that a junk-food diet can increase impulsivity on a variety of behavioral tests, including delay discounting (5), vigilance (6), open field (66), and reversal learning (67), an effect that may be passed on to offspring as a result of an “unfavorable intrauterine nutritional environment” (68). The indiscriminant food seeking exhibited by rats in the junk-food groups may reflect a lack of top-down cognitive control over behavior. Indeed, it was recently shown that a refined, obesogenic diet (modeling processed foods) can increase premature responding and disrupt attentional processes in a vigilance task (6). Furthermore, high-fat, high-carbohydrate diets have been shown to impair reversal learning, resulting in perseverative responding following a change in reinforcement contingencies, suggesting a decreased capacity to either inhibit incorrect responding or appropriately use cue-outcome associations to guide behavior (67).

Surprisingly, we found no difference in behavior between rats exposed to junk food on an intermittent vs. *ad libitum* schedule on either measure, despite significant differences in total and binge junk-food consumption and differences in behavior reported in other studies (i.e., intermittent exposure tends to produce a behavioral phenotype akin to addiction, while *ad libitum*, 24-h exposure does not) (14, 32, 33, 36, 69). This suggests that the causal factor for the deficits reported here is access to junk food *per se*, as both patterns of exposure provide a complex sensory experience and potential for physiological changes. However, to our knowledge, there are few within-study comparisons between intermittent and *ad libitum* junk-food exposure. Direct comparisons between exposure patterns across studies should be approached with caution since they may be due to other methodological differences. It is also possible that all junk food-exposed rats in our study experienced a “binge” effect of sorts during daily food changes, when old foods were removed and fresh foods were provided, creating a pseudo-binge-like phenotype in even the *ad libitum*-exposed rats, minimizing differences between each group. Importantly, given that rats were pair-housed during junk-food exposure, we did not have sufficient power to evaluate whether individual differences in junk-food consumption correlated with diet-induced behavioral deficits. Furthermore, although exploratory correlational analyses did not find any evidence for a relationship between final body weight or diet-induced weight gain and the severity of impairment during devaluation or PIT testing (data not shown), such analyses also lacked sufficient power to draw firm conclusions one way or the other. Thus, although we suggest that the behavioral deficits reported here likely relate to the psychological experience of repeatedly consuming palatable junk food, it remains unclear whether such effects are (also) driven by diet-induced changes in body composition and/or metabolism.

In summary, we found that access to a junk-food diet *following* training impaired the ability of rats to adjust their food-seeking actions in response to either satiety-induced devaluation of reward or cue-triggered food expectations. These and related findings indicate that repeated access to such diets can lead to long-lasting changes in behavioral control which may contribute to overeating and obesity. Our findings suggest that while junk-food exposure does not lead to exaggerated levels of food seeking, it does cause a dysregulation of food-motivated behavior, which may lead to indiscriminant, or mindless, food grazing.

## Author contributions

AK conducted the experiments with assistance from JA, LT, GC. Experimental design, data analysis and interpretation, and writing were done by AK, NTM, NPM, and SO.

### Conflict of interest statement

The authors declare that the research was conducted in the absence of any commercial or financial relationships that could be construed as a potential conflict of interest.
